# Adequacy of Nutritional Intakes during the Year after Critical Illness: An Observational Study in a Post-ICU Follow-Up Clinic

**DOI:** 10.3390/nu14183797

**Published:** 2022-09-15

**Authors:** Anne-Françoise Rousseau, Sara Lucania, Marjorie Fadeur, Anne-Marie Verbrugge, Etienne Cavalier, Camille Colson, Benoit Misset

**Affiliations:** 1 Intensive Care Department, University Hospital of Liège, University of Liège, 4000 Liège, Belgium; 2 Multidisciplinary Nutrition Team, University Hospital of Liège, 4000 Liège, Belgium; 3 Clinical Chemistry Department, University Hospital of Liège, University of Liège, 4000 Liège, Belgium

**Keywords:** nutrition, protein, energy, oral nutrition, dietary assessment, nutrition intake, critical illness, survivors

## Abstract

Whether nutritional intakes in critically ill survivors after hospital discharge are adequate is unknown. The aims of this observational study were to describe the energy and protein intakes in ICU survivors attending a follow-up clinic compared to empirical targets and to explore differences in outcomes according to intake adequacy. All adult survivors who attended the follow-up clinic at 1, 3 and 12 months (M1, M3, M12) after a stay in our intensive care unit (ICU) ≥ 7 days were recruited. Average energy and protein intakes over the 7 days before the face-to-face consultation were quantified by a dietician using food anamnesis. Self-reported intakes were compared empirically to targets for healthy people (FAO/WHO/UNU equations), for critically ill patients (25 kcal/kg/day and 1.3 g protein/kg/day). They were also compared to targets that are supposed to fit post-ICU patients (35 kcal/kg/day and 1.5 g protein/kg/day). Blood prealbumin level and handgrip strength were also measured at each timepoint. A total of 206 patients were analyzed (49, 97 and 60 at the M1, M3 and M12, respectively). At M1, M3 and M12, energy intakes were 73.2 [63.3–86.3]%, 79.3 [69.3–89.3]% and 82.7 [70.6–93.7]% of healthy targets (*p* = 0.074), respectively. Protein intakes were below 0.8 g/kg/day in 18/49 (36.7%), 25/97 (25.8%) and 8/60 (13.3%) of the patients at M1, M3 and M12, respectively (*p* = 0.018), and the protein intakes were 67.9 [46.5–95.8]%, 68.5 [48.8–99.3]% and 71.7 [44.9–95.1]% of the post-ICU targets (*p* = 0.138), respectively. Prealbumin concentrations and handgrip strength were similar in patients with either inadequate energy intakes or inadequate protein intakes, respectively. In our post-ICU cohort, up to one year after discharge, energy and protein intakes were below the targets that are supposed to fit ICU survivors in recovery phase.

## 1. Introduction

Survivors of critical illness report several functional impairments that can persist in the months after discharge. Some of these impairments can be related to intensive care unit (ICU)-acquired weakness. Muscle wasting is part of this deficit. Nutritional strategies could play an important role in improving muscle mass recovery [1]. The existing literature about nutritional care in critically ill patients is mainly centered on the acute phase, and optimal quantity and quality of energy and protein intakes are still poorly defined in ICU survivors. In the period of convalescence and rehabilitation, higher energy and protein requirements may be assumed to face persistent catabolism and secondary anabolism. According to experts, energy intakes could be increased to 150% of the predicted requirements or to provide 35 kcal/kg/day, while protein intakes could even be higher than 2 g/kg/day [2]. However, these targets have not been validated in post-ICU studies directly measuring energy expenditure or protein loss, nor in studies reporting outcomes associated with increased intakes.

After ICU discharge, oral nutrition is the most common mode of nutrition provision after ICU discharge [3]. However, this route exposes patients to the highest risk of nutritional deficiencies. Barriers to adequate nutritional intakes in the post-ICU period are related to reduced appetite, taste changes, chewing difficulties, swallowing impairment or cognitive disorders and inadequate food services. Some previous reports confirm this issue, describing inadequate intakes in patients on an oral route after mechanical ventilation liberation [4,5], as during hospitalization after ICU discharge [6,7]. The transition between the hospital ward and home is often a challenging period, characterized by communication gaps, fragmentation of care and omissions in treatment [8]. The issues of ICU survivorship are complex but still poorly addressed outside multidisciplinary post-ICU follow-up clinics [9]. Few survivors have access to such specialized support and must rely on primary care. In such contexts, and if nutritional targets are so much higher than hypothesized, the risk of inadequate nutritional intakes is a real concern. However, little is currently published to inform on the nutritional intakes in ICU survivors after hospital discharge.

Considering the negative impact of the post-ICU survivorship on patient’s quality of life [10] and healthcare costs [11], the post-ICU phase has become a research priority, especially from a nutrition point of view [12]. The primary aim of the present observational study was to describe the energy and protein intakes of ICU survivors attending a follow-up clinic. In the absence of specific guidelines for the post-ICU period, the observed intakes were compared to different empirical targets dedicated to healthy patients, ICU patients or post-ICU survivors. The secondary objective was to explore differences in outcomes according to intake adequacy.

## 2. Materials and Methods

### 2.1. Population

Patients surviving an ICU stay ≥ 7 days are systematically invited to our post- intensive care follow-up clinic, at 1-, 3- and 12-months following ICU discharge. Patients do not enter the post-ICU trajectory of our follow-up clinic if they are unable to communicate in French (the local language), if they have been transferred to another hospital or if we are unable to give them information about the clinic. The scheduled face-to-face consultation is generally canceled if they are still hospitalized in an acute care facility or in an inpatient rehabilitation facility, or if they refuse it. A dedicated multidisciplinary team, including critical care physicians, critical care nurses (coordinators of the clinic), physiotherapists, dieticians and psychologists is involved at each timepoint. The follow-up is standardized, addressing physical status and functional performances, nutritional status and body composition, bone health, mental health disorders, cognitive impairment, sleep disorders and health-related quality of life. A blood analysis focuses on inflammation and metabolic biomarkers. Organ-specific assessments are not managed by the follow-up clinic, but rather by the referent specialists. In case PICS-related problems are detected, direct advice is provided (i.e., physical activity, sleep hygiene education), medications are prescribed as needed (i.e., antalgic treatment, melatonin) or patients are referred to specialists according to the symptoms (i.e., psychologist, rehabilitation specialist, neuropsychologist, geriatrician). More recently, we included a screening of socioeconomic problems in the standardized follow-up (debts, lack of health insurance coverage). Patients are referred to social workers if needed.

The dietician consultation lasts 20 to 30 min, resulting in general nutritional advice. Unfortunately, there is not enough time for the provision of a personalized meal plan.

Over 14 months, from 1 January 2021 to 29 February 2022, all consecutive critically ill survivors who attended our follow-up clinic consultation at 1, 3 and/or 12 months after discharge (respectively M1, M3, M12) were enrolled in the present observational study. Patients were further excluded if they did not benefit from the dietician consultation or in case of missing nutritional data. Patients who attended two consultations were analyzed separately.

In accordance with Belgian law, informed consent was not required because the study did not modify patients’ management and the data were anonymously collected. This interpretation was confirmed by the Ethics Committee of the University Hospital of Liege (local reference 2020/424).

### 2.2. Assessment of Nutritional Intakes

Food anamnesis included a semistructured food consumption survey and a food frequency questionnaire, focused on the 7 days before the visit. Photographs of utensils and containers were used to help quantify serving sizes. The energy and protein average intakes were determined: the foods were converted into nutrients using the Belgian Food composition database (Brussels, Belgium) (6th Edition, 2017: http://www.nubel.com/fr/table-de-composition-des-aliments.html) (accessed on 1 January 2021). In case some foods were not listed in the Belgian database, the French food composition database (https://ciqual.anses.fr) (accessed on on 1 January 2021) was used.

### 2.3. Calculation of Nutritional Targets

Three types of targets were chosen. First, targets for healthy people were calculated using the recommendations of the Joint FAO/WHO/UNU Expert Consultation (https://www.fao.org/3/y5686e/y5686e00.htm#Contents (accessed on 1 July 2022) and https://apps.who.int/iris/handle/10665/43411) (accessed on 1 July 2022). Daily protein requirements were defined as 0.8 g/kg. The basal metabolic rate (BMR) was calculated using predictive equations based on age, sex, weight (W, in kilograms) and height (H, in meters): male 18–30 years—15.4 × W – 27 × H + 717; male 30–60 years—11.3 × W – 16 × H + 901; male >60 years—8.8 × W + 1128 × H – 1071; female 18–30 years—13.3 × W + 334 × H + 35; female 30–60 years—8.7 × W – 25 × H + 865; female >60 years—9.2 × W + 637 × H – 302. The total energy expenditure (considered as daily caloric requirements) was then calculated by multiplying BMR by the physical activity level (PAL). PAL is a number expressing the patient’s daily physical activity: 1.53 if sedentary or light activity lifestyle, 1.76 if active or moderately active lifestyle, 2.25 if vigorously active lifestyle. Physical activity level was remotely quantified using the French version of the International Physical Activity Questionnaire-Short Form (IPAQ-SF). Patients were asked to report their typical weekly activity types, including vigorous-intensity activities (e.g. heavy lifting, digging, aerobics, or fast bicycling); moderate-intensity activities (e.g. carrying light loads, bicycling at a regular pace, or doubles tennis); walking; and sitting that is undertaken during work, transport, housework, or leisure activities. The total score is the summation of the duration and frequency of walking and moderate- and vigorous-intensity activities, reported as the “metabolic equivalent of task-min per week (MET-minute/week)” [13]. The IPAQ-SF scores of <600, 600–3000 and >3000 MET-min/week were respectively categorized as low, moderate and high physical activity levels according to the guidelines of the IPAQ (https://sites.google.com/site/theipaq/scoring-protocol) (accessed on 1 July 2022).

Second, targets for critically ill patients were calculated according to the latest version of the ESPEN guidelines on clinical nutrition in the intensive care unit [14]. Daily energy and protein requirements were defined as 25 kcal/kg and 1.3 g/kg, respectively. In obese patients without chronic kidney disease, protein target was considered as 2 g/kg/day.

Third, targets for patients in post-ICU phase were calculated according to practical guidance from experts in the field, stating that higher energy and protein intakes could be required for recovery over months to years in patients who have lost significant muscle mass and strength following an ICU stay [2,15]. Daily energy and protein requirements were considered as 35 kcal/kg and 1.5 g/kg, respectively. In obese patients without chronic kidney disease, protein targets were considered as 2 g/kg/day.

In case of chronic kidney disease in stage 3 to 5 (i.e., estimated glomerular filtration rate <60 mL/min/1.73 m^2^), protein targets were considered as 0.8 g/kg/day according to latest dedicated nutritional guidelines [16].

### 2.4. Other Clinical Data

Demographic data (age, sex, weight before ICU admission, weight during the first week after ICU discharge and actual weight at the time of consultation either measured or obtained from patient’s report), height, body mass index (BMI)) were recorded. Actual weight was used for patients with BMI <25 kg/m^2^. Ideal body weight (IBW) was considered as the expected weight for BMI 25 in overweight patients. In obese patients (BMI ≥ 30), adjusted body weight was calculated as follows: IBW  +  0.33 × (actual weight − IBW) [14].

Data about the ICU stay were also recorded.

At the time of the consultation, patients were asked about any loss of appetite and swallowing problems. The risk of malnutrition was assessed using the “Malnutrition Universal Screening Tool” (MUST), a validated tool for outpatients [17]. The score is based upon BMI, a history of recent weight loss, and the effect of acute disease. In this cohort, we considered the acute disease score as 0, as there was unlikely to be no nutritional intake for more than 5 days in the context of outpatient post-ICU follow-up. A MUST score of 0, 1 or ≥2 indicates a low, moderate or high risk of malnutrition, respectively. The diagnosis of malnutrition was made using the Global Leadership Initiative on malnutrition (GLIM) [18]. We considered that an etiologic criterion was always present at M1 (i.e., reduced food intake). Phenotypic criteria were assessed using BMI and % weight loss. Moderate malnutrition was defined by weight loss between 5 and 10% within the past 6 months or between 10 and 20% beyond 6 months and/or BMI < 20 kg/m^2^ (if age <70 years) or BMI < 22 kg/m^2^ (if age ≥70). Severe malnutrition was defined by weight loss over 10% within the past 6 months or over 20% beyond 6 months and/or BMI < 18.5 kg/m^2^ (if age <70 years) or BMI < 20 kg/m^2^ (if age ≥70).

Total body fat was measured using bioelectrical impedance with Bodystat Quadscan 4000 at a frequency of 50 Hz (Bodystat, Douglas, Ilse of Man, UK). Data in men and women were compared to body composition of healthy Caucasian adults [19].

At each timepoint, functional outcomes were assessed. Activities of daily living were assessed using Barthel Index, a questionnaire measuring functional status and dependency. It consists of 10 subheadings, namely feeding, bathing, grooming, dressing, bladder control, bowel control, toilet use, chair–bed transfer, mobility and stair climbing [20]. Scoring ranges from 0–100: a score of 100 is defined as being capable of ADL complete self-care. Handgrip strength was assessed using Jamar hydraulic hand dynamometry. Measurements were performed in a sitting position, with the elbow in 90° flexion. The protocol consisted of three consecutive maximal contractions for each muscle group, preceded by three warm-up trials. Observers provided standardized encouragement. The three measurements were performed with 30 s intervals between contractions. Subjects were asked to gradually increase their muscle force to a maximum effort, which had to be sustained for 6-s. The highest performance was considered for analysis.

### 2.5. Biological Data

The biological data were generated from one single laboratory (Unilab, University Hospital of Liège, Belgium) accredited for ISO 15,189 guideline. Blood samples were collected during the afternoon, in a non-fasting status. Blood levels of albumin were assayed by spectrophotometry (Alinity C, Abbott, Chicago, IL, USA). Blood prealbumin and C-reactive protein concentrations were assayed using immunoturbidimetry (Alinity C, Abbott, Chicago, IL, USA). Blood levels of creatinine and triglycerides were assayed using an enzymatic assay (Alinity C, Abbott, Chicago, IL, USA). Blood levels of calcidiol (25OH-D) were measured using an immunoassay based on chemiluminescence (CLIA) (Liaison XL^®^, DiaSorin, Stillwater, MN, USA). The glomerular filtration rate was estimated using creatinine-based CKD-EPI equation.

### 2.6. Study Outcomes and Analysis

The primary objective of this study was to assess energy and protein intake expressed as a percentage of calculated targets (adequacy) between patients at M1, M3 and M12. The secondary objective was to explore differences in outcomes according to intake adequacy. Adequacy was based on achieving 100% or more of the targets for healthy people.

Patients who attended more than one consultation were analyzed separately.

The nutritional intakes of older adults (i.e., ≥60 years, according to the WHO definition) were compared to those of younger patients.

### 2.7. Statistical Analysis

Statistical analysis was performed using Graphpad Prism (version 6.0 for Mac OSX, Graphpad Inc., San Diego, CA, USA). Normality was assessed using the Shapiro–Wilk test. As some datasets did not pass the normality test, the results were expressed as medians with lower and upper quartiles [Q1–Q3] for quantitative parameters, or as counts and proportions for qualitative parameters. Comparisons between unpaired data were made using the Mann–Whitney test. Comparisons between paired data were made using the Wilcoxon test. A chi-squared test was used to compare categorical variables. A *p* value < 0.05 was considered statistically significant.

## 3. Results

### 3.1. Patients

**Figure 1:**
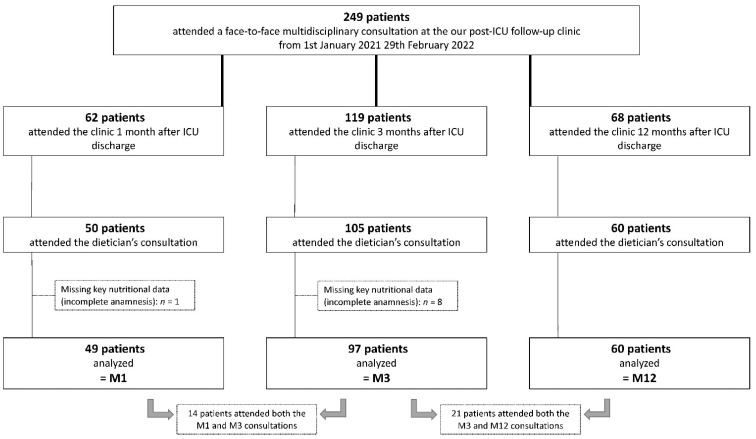
Flow chart.

From 1 January 2021 to 29 February 2022, 249 patients attended the post-ICU follow-up clinic. A small proportion of these patients could not be included in the present study because they did not attend the consultation with the dietitian or because some nutritional data were missing. Finally, 49, 97 and 60 patients were analyzed from the M1, M3 and M12 consultation, respectively (Figure 1).

The characteristics of the patients included at the three timepoints are detailed in Table 1. At M1, M3 and M12, 2/49 (4.1%), 6/97 (6.2%) and 1/60 (1.7%) patients were ≥80 years old, respectively. Patients from the M12 consultation were mainly survivors of a critical COVID-19 pneumonia. Only a small proportion of patients were referred to social workers for socioeconomic problems at the three timepoints (*p* = 0.932).

### 3.2. Nutritional Data

The nutritional-related data at each timepoint are described in Table 2. Weight measured at the consultation was significantly lower than the pre-ICU body weight at M1 (*p* = 0.002) and M3 (*p* < 0.001). However, at M12, actual weight was similar to pre-ICU weight (*p* = 0.132). Body composition was abnormal at the three timepoints; fat mass in both males and females were higher than normal values for healthy adults. A higher proportion of patients at high risk of malnutrition (MUST ≥ 2) was observed at M1, compared to M3 and M12 (*p* = 0.034). The proportion of patients with moderate or severe malnutrition based on the GLIM criteria significantly decreased over time (both *p* < 0.001). All the patients were fed by oral route. Dysphagia was suspected in less than 15% of the patients. The proportion of patients who declared a loss of appetite was higher at M1 (18/49, 36.7%) compared to M3 (29/97, 30%) and M12 (7/60, 11.6%) (*p* = 0.004).

Blood biomarkers were into normal ranges at the three timepoints (Table 3). A similar proportion of CKD stage 3 to 5 was observed at M1 (14/49, 28.6%), at M3 (18/97, 18.6%) and at M12 (11/60, 18.3%) (*p* = 0.315).

Energy and protein intakes and targets are detailed in Table 2. A high proportion of patients had energy intakes under the healthy target (FAO/WHO/UNU equations) in M1: 43/49 (87.8%). This proportion was similar at M3 (88/97, 90.7%) and at M12 (53/60, 88.3%) (*p* = 0.387). Energy intakes reached similar percentages of the healthy target at the three timepoints (*p* = 0.074). Compared to ICU targets (25 kcal/kg/day), about half of the patients had lower energy intakes (27/49, 55%) at M1. This proportion tended to decrease at M3 (39/97, 40.2%) and M12 (20/60, 33.3%), but without a significant difference (*p* = 0.067). A majority of patients had energy intakes below the post-ICU target (35 kcal/kg/day): 45/49 (91.8%) at M1, 94/97 (97%) at M3 and 58/60 (96.7%) at M12 (*p* = 0.33). Regarding proteins, the proportion of patients with intakes below the healthy target (0.8 g/kg/day) was higher at M1 (18/49, 36.7%) compared to M3 (25/97, 25.8%) and M12 (8/60, 13.3%) (*p* = 0.018). Protein intakes reached higher percentages of healthy targets at M3 and M12 compared to M1 (*p* = 0.042), while they reached similar percentages of post-ICU targets at the three timepoints (*p* = 0.138).

### 3.3. Outcome Analysis According to Nutritional Adequacy

Prealbumin level and handgrip strength in patients with respective energy and protein intakes below healthy targets were compared to levels in patients who reached these targets (Table 4). No difference in blood prealbumin level was observed at each timepoint whether energy intakes reached the targets or not. Except for in males at M1, handgrip strength was not lower in patients whose protein intakes reached targets compared to patients with protein adequacy.

### 3.4. Nutritional Data in Patients Who Attended More Than One Consultation

A total of 14 and 21 patients attended both M1 and M3 or M3 and M12 consultations, respectively. Their nutritional data are detailed in Table 5. Patients gained weight either between M1 and M3 consultations or between M3 and M12 consultations (both *p* = 0.004). However, their energy and protein intakes were stable between two consultations.

### 3.5. Nutritional Intakes in Older Adults Compared to Younger Patients

There were 25/60, 59/97 and 37/60 older patients at M1, M3 and M12, respectively. The nutritional intakes in older patients compared to younger patients are detailed in Table S1. The energy and protein intakes, expressed as percentages of the respective targets, were similar in both subgroups, at the three timepoints.

## 4. Discussion

In this study, we investigated nutritional intakes in a large number of ICU survivors with a prolonged ICU stay, up to one year after ICU discharge. Self-reported energy intakes at the M1, M3 and M12 consultations were lower than the empirically chosen targets in a significant proportion of patients: at least 80% of the patients reported energy intakes below the target for healthy individuals (based on the FAO/WHO/UNU equations). Protein intakes lower than 0.8 g/kg/day (the considered target for healthy individuals) were observed in one-third of the patients in the early post-ICU trajectory and became less frequent at M3 and M12. Age did not seem to influence the percentage of energy or protein targets reached by the nutritional intakes.

Previously, two studies with a low number of patients pointed out that reported nutritional intakes three months after an ICU stay could be below estimated requirements [21] or inferior to those of healthy patients [22]. A poor appetite may influence dietary intakes [23]. As in other previous studies [22,24], a loss of appetite was observed in at least one third of the survivors one month after discharge and seemed to improve during the following months. In parallel, one year after ICU discharge, survivors regained their pre-ICU weight, despite apparent nutritional inadequacy. An abnormal body composition was also observed, suggesting an altered lipid metabolism. Long-term metabolic disturbances have already been demonstrated after severe burn injury [25], but this is less described after a general critical illness.

The nutritional requirements for ICU survivors are thought to be higher than healthy patients, but they are still not defined [2]. In the present study, different theoretical targets related to different clinical status were calculated. Assuming that ICU survivors would require 35 kcal/kg/day of energy, their intakes would be close to the requirements of healthy patients according to the FAO/WHO/UNU equations. In the very near future, clinical investigations should aim to determine the actual ranges of requirements, at least the energy requirements. This is now feasible using the new generation of indirect calorimeter, allowing for an easy and accurate measurement of energy expenditure in spontaneously breathing patients [26,27]. The task will probably be more complex for protein requirements.

Less than 10% of the studied patients were considered at risk of malnutrition, mainly during the first three months following an ICU stay. A diagnosis of malnutrition was made in half of the studied population at M1 but was rare at M12. Screening or diagnostic tools of malnutrition include disease burden or inflammation as one of their criteria [28]. It is unclear whether ICU survivors, months after the ICU stay, fit this criterion or not. As observed in the present study, blood biomarkers related to nutritional status, including CRP, are into normal ranges in most of ICU survivors. However, some data indicate a persistent low-grade inflammation associated with an oxidative stress [29]. Taking all growing evidence into account, it seems reasonable to consider all ICU survivors as at persistent risk of malnutrition, at least up to 3 months after discharge.

Most of the studies observing the impact of nutritional strategies on the subsequent recovery were performed during the acute phase. Non-individualized approaches and inadequate outcomes may explain some of the observed negative results. It is noteworthy that nutrition-related concerns persist after the removal of the immobilization or critical stimuli. Optimal nutritional support after critical illness is often considered from a quantitative point of view. However, quality of nutritional intakes could probably also make a difference: some specific macronutrients and micronutrients can modulate muscle anabolism, inflammation or mitochondrial function [30,31,32]. To date, the impact on outcomes of enhanced nutritional strategies aiming at increasing energy, protein, specific protein or micronutrient intakes during the post-ICU phase is unknown. In the present study, inadequate energy or protein intakes were not associated with lower levels of prealbumin or weaker handgrip strength, respectively. However, this study was not designed for outcome assessment, and specific macro- or micronutrient intakes were not quantified. Further investigations should use targeted food anamnesis and dedicated core outcome sets such as the novel one recently developed for nutrition studies [33].

The present observations should help to raise awareness of the post-ICU nutritional concerns among secondary and primary care providers, who are not familiar with the post-ICU syndrome [34]. They also highlight the need for a closer and regular nutritional follow-up, at least during the first months following hospital discharge. This could allow for the initiation of appropriate interventions to improve nutritional intakes. Following the “SPICES concept” [15], different points should be addressed to optimize oral feeding: swallowing disorder screening and management, patient status overview aiming at detecting the elements of the post-ICU syndrome [35], dietician and nutritionist involvement, and supplementation in macro- and micronutrients. Nutritional targets may be found to be enormous by ICU survivors compared to the amounts they are able to ingest. In order to achieve macronutrient targets when orally fed, food enrichment or oral nutrition supplements (ONS) will often need to be considered. In different systematic reviews and meta-analysis in adults of various medical backgrounds [36,37,38], ONSs were associated with improvements in nutritional intakes, body composition or grip strength. They also have been shown to have a positive impact on hospital readmission rates. To date, the benefits of ONSs in the specific post-ICU population have not been investigated yet. To date, in the absence of specific guidelines on macro- and micronutrients requirements for ICU survivors, it is not possible to make any suggestions based on the present results in terms of amounts to be supplemented. However, nutritional support needs to be individualized, taking into account the global health condition of the patients, in addition to their post-ICU condition.

Some limitations need to be acknowledged. First, nutritional intakes were self-reported and not recorded daily using an objective method such as pictures of meals. Results of nutrition surveys may thus be biased due to the fact that participants, consciously or unconsciously, forgot to declare some foods or misjudged amounts. These biases are unfortunately inherent to the method. Second, swallowing disorders and appetite loss were screened using open questions only. Our post-ICU consultations aim for a comprehensive assessment of different domains of the post-ICU syndrome. They last about 2 h. In such a model, it is difficult to perform objective and detailed screening for all domains, and to multiplicate the tests used. Third, the study was monocentric and relied on patients who attended an in-hospital consultation. Some of our local ICU survivors did not attend our clinic; some refused, either because they had no complaints or on the other hand, because they were bedridden. Others were lost at follow-up due to reduced human resources in our follow-up clinic. The present results could thus not reflect the nutritional status of all ICU survivors. However, this pragmatic study is an audit of patients’ nutritional conditions in the real life of a post-ICU follow-up clinic. Fourth, medical history was not considered when analyzing the reported nutritional intakes. Nutritional needs are influenced by many parameters, underlying the importance of an individualized approach. Fifth, the number of very old patients (i.e., ≥80 years) was very low at the three timepoints, precluding any comparison with younger patients. However, very old patients are probably those who could experience the more severe nutritional problems in terms of low intakes and undernutrition. Analyzing this category of patients in further studies would be relevant. Finally, some patients attended more than one consultation and were then included in more than one group in order to depict the nutritional conditions and needs at each timepoint. This way of analyzing could have impacted the observations, especially if they implemented the dietary advice received during their first consultation. However, in patients who attended two consecutive consultations, no differences in energy or protein intakes were observed between the two timepoints. General dietary advice may be difficult to accept or apply, underlying the need for a multidisciplinary nutritional plan and regular dietician follow-up.

## 5. Conclusions

In ICU survivors attending our post-ICU follow-up clinic, up to one year after discharge, energy and protein intakes were below the targets supposed to fit ICU survivors in the recovery phase. Compared to targets for healthy people, energy intakes were considered inadequate in the majority of patients, while protein inadequacy was less frequent and gradually improved. These observations highlight the need for a close and prolonged nutritional follow-up, independently of patients’ age, ideally involving dieticians and nutritionists.

## Figures and Tables

**Table 1:** Patients’ characteristics at the three timepoints.

Data		M1 *n* = 49	M3 *n* = 97	M12 *n* = 60
Age, year		60 [51–67]	62 [54–70]	61.5 [52–68]
Male, n (%)		32 (65.3)	59 (60.8)	41 (68.3)
SAPS II		28.5 [24–39.5]	31 [25–49]	34 [25.5–49]
Admission failure, *n* (%)	Cardiovascular	22 (44.9)	28 (28.9)	6 (10)
	Pulmonary	21 (42.9)	49 (50.5)	51 (85)
	Neurologic	3 (6.1)	9 (9.3)	2 (3.3)
	Digestive	1 (2)	2 (2.1)	0
	Trauma	0	1 (1)	0
	Other	2 (4.1)	8 (8.2)	1 (1.7)
Mechanical ventilation, n (%)		24 (49)	57 (58.8)	47 (78.3)
Duration of mechanical ventilation, day		2 [1–7]	8 [2.5–19.5]	13 [8–24]
ICU LOS, day		7.5 [7–10]	11 [8–20]	16 [11–27]
Hospital LOS, day		21.5 [13.5–38.8]	30 [19–51]	34.5 [22–50.2]
Socioeconomic problems, n (%)		2/31 (6.5)	3/63 (4.8)	2/42 (4.8)

Data are expressed as median with lower and upper quartiles [Q1–Q3]. ICU: intensive care unit; LOS: length of stay; SAPS II: Simplified Acute Physiology Score.

**Table 2:** Nutritional data, body composition and functional outcomes at the three timepoints.

Data		M1 *n* = 49	M3 *n* = 97	M12 *n* = 60
Initial weight, kg		88 [69–102]	84 [70–95]	92 [74.5–105]
Weight during the week after ICU discharge, kg		79.5 [67.3–92.8]	71.9 [62–86]	81.8 [64.5–90.4]
Actual weight, kg		81.5 [66.2–93.5]	77 [67.5–90]	90 [76.2–101]
Actual weight considered for nutritional calculation, kg		77.1 [66.2–84.5]	73 [65.2–82]	79.1 [68.7–84.3]
BMI, kg/m^2^		26.6 [22.7–30.9]	26.7 [23.7–31.1]	31.6 [26.8–34.2]
Fat mass, % of total body weight	Males	27.5 [24.3–32.8]	28.6 [24.7–32.4]	28.7 [23.5–32.4]
	Females	41.4 [29.1–48.4]	41.6 [34.4–45.6]	38.8 [33.5–43.6]
MUST score ≥ 2, *n* (%)		5 (10.2)	4 (4.1)	0
GLIM, *n* (%)	Moderate malnutrition	16 (32.7)	5 (5.2)	1 (1.7)
	Severe malnutrition	11 (22.4)	5 (5.2)	0
Swallowing problems, *n* (%)		3 (6.1)	14 (14.4)	5 (8.3)
Loss of appetite, *n* (%)		18 (36.7)	29 (30)	7 (11.6)
Energy intakes, kcal/day		1800 [1530–2250]	2000 [1619–2200]	2100 (1778–2400]
Energy intakes, kcal/kg/day		24.5 [21.2–29.3]	26.1 [23–29.7]	27 [23.1–29.1]
Energy healthy target (FAO/WHO/UNU), kcal/day		2518 [2238–2810]	2392 [2169–2756]	2425 [2239–2763]
Energy intakes, % of healthy target		73.2 [63.3–86.3]	79.3 [69.3–89.3]	82.7 [70.6–93.7]
Energy ICU target (25 kcal/kg/day), kcal/day		1928 [1656–2113]	1825 [1631–2050]	1980 [1735–2139]
Energy post-ICU target (35 kcal/kg/day), kcal/day		2699 [2319–2958]	2555 [2284–2870]	2772 [2428–2995]
Protein intakes, g/day		70 [50–87.4]	80 [60–90]	86.2 [68.9–110]
Protein intakes, g/kg/day		0.94 [0.7–1.22]	1.07 [0.8–1.2]	1.11 [0.9–1.33]
Protein healthy target (0.8 g/kg/day), g/day		61.7 [53–67.6]	58.4 [52.2–65.6]	63.4 [55.4–68.8]
Protein intakes, % of healthy target		117.6 [87.2–152.3]	134.1 [99.7–155.7]	134.6 [109.9–158.2]
Protein ICU target (1.3 g/kg/day or 2 g/kg/day if obese, or 0.8 g/kg/day if CKD), g/day		91.2 [70.2–122.2]	92.6 [74.1–132.5]	123.3 [83.4–164.1]
Protein post-ICU target (1.5 g/kg/day or 2 g/kg/day if obese or 0.8 g/kg/day if CKD), g/day		99.7 [74.4–137.2]	106.8 [85.5–139.5]	136.8 [94.7–164.1]
Protein intakes, % of post-ICU target		67.9 [46.5–95.8]	68.5 [48.8–99.3]	71.7 [44.9–95.1]
Barthel index		100 [100–100]	100 [100–100]	100 [100–100]
Handgrip strength, kg	Males	33.5 [29.7–41]	35 [29–42]	36 [25–43.5]
	Females	22 [18.5–25.8]	19 [14–24]	33.5 [21.5–43]

Data are expressed as median with lower and upper quartiles [Q1–Q3]. CKD: chronic kidney disease; ICU: intensive care unit.

**Table 3:** Biological parameters at the three timepoints.

Blood Analysis	Reference Ranges	M1 *n* = 49	M3 *n* = 97	M12 *n* = 60
C-reactive protein (CRP), mg/L	0–5	3.1 [1.6–19.6]	2.5 [1–5.1]	2.4 [1.2–4.6]
Albumin, g/L	≤ 60 years: 35–52 >60 years: 32–46	41 [39–43]	43 [41–44]	44 [43–46]
Prealbumin, g/L	0.2–0.4	0.29 [0.24–0.34]	0.28 [0.25–0.32]	0.3 [0.25–0.34]
Triglycerides, mg/dL	<175	163 [124–202.5]	163 [113.8–221.8]	172.5 [108–244.8]
25OH-D, ng/mL	20–50	25.7 [16.4–37.7]	30 [20.7–37.6]	26.8 [17.5–33.9]
Creatinine, mg/dL	Males: 0.55–1.18 Females: 0.55–1.02	0.93 [0.71–1.32]	0.91 [0.71–1.11]	0.99 [0.82–1.14]

Data are expressed as median with lower and upper quartiles [Q1–Q3].

**Table 4:** Differences in outcomes (prealbumin and handgrip strength) according to nutritional intake adequacy based on healthy targets.

Prealbumin, g/L				
**Timepoints**		**Energy intakes < healthy targets (FAO/WHO/UNU)**	**Energy intakes ≥ healthy targets (FAO/WHO/UNU)**	***p* value**
M1		0.29 [0.24–0.34]	0.31 [0.19–0.4]	0.815
M3		0.28 [0.25–0.32]	0.26 [0.18–0.36]	0.554
M12		0.3 [0.25–0.34]	0.33 [0.24–0.36]	0.776
**Handgrip Strength, kg**				
**Timepoints**		**Protein intakes < healthy targets (0.8 g/kg/day)**	**Protein intakes ≥ healthy targets (0.8 g/kg/day)**	***p* value**
M1	Males	30 [21.7–33]	39 [33–43.3]	0.003
	Females	23.5 [18.7–27.5]	22 [20–24]	0.553
M3	Males	32 [24–42]	36 [29.5–42]	0.585
	Females	18 [14–20]	20.5 [16.2–25.5]	0.091
M12	Males	35.5 [25.2–37.5]	38 [25–48]	0.441
	Females	26 [18–43]	34 [22–42]	0.657

Data are expressed as median with lower and upper quartiles [Q1–Q3].

**Table 5:** Evolution of weight and nutritional intakes over time in patients who attended more than one follow-up consultation.

Data	Patients Who Attended Both M1 and M3 Consultations (*n* = 14)		*p* Value
	**M1**	**M3**	
Actual weight, kg	82.7 [60.2–88.5]	86.5 [66.2–91.4]	0.004
Energy intakes, kcal/day	1710 [1325–1975]	2000 [1570–2165]	0.064
Protein intakes, g/day	65 [42.5–82.2]	73.9 [54.9–83.7]	0.445
	**Patients Who Attended Both M3 and M12 Consultations (n = 21)**		
	**M3**	**M12**	
Actual weight, kg	79 [69–90.8]	79.8 [68–97.5]	0.004
Energy intakes, kcal/day	2000 [1800–2275]	2045 [1787–2436]	0.12
Protein intakes, g/day	80 [52.5–90]	80.1 [60.4–107.5]	0.056

Data are expressed as median with lower and upper quartiles [Q1–Q3].

## Data Availability

The datasets used and/or analyzed during the current study are available from the corresponding author on reasonable request.

## Supplementary Materials

The following supporting information can be downloaded at: https://www.mdpi.com/article/10.3390/nu14183797/s1, Table S1: Nutritional data at the three timepoints, according to age.
