# Supplementary material for: Adequacy of Nutritional Intakes during the Year after Critical Illness: An Observational Study in a Post-ICU Follow-Up Clinic
Source: Nutrients. 2022 Sep 15;14(18):3797. doi: 10.3390/nu14183797 (PMC9502764; doi:10.3390/nu14183797)
Supplement: Supplementary file 1 [file nutrients-14-03797-s001.zip › nutrients-1900871-supplementary.pdf]

Table S1. Nutritional data at the three timepoints, according to age.

Data	M1 *n*=49		M3 *n*=97		M12 *n*=60	
	**< 60 years** *n*= 24	**≥ 60 years** *n*= 25	**< 60 years** *n*= 38	**≥ 60 years** *n*=59	**< 60 years** *n*=23	**≥ 60 years** *n*=37
Actual weight, kg	79.2 [64.2-92.8]	83 [66.5-95]	76 [62.3-90.5]	77 [69-90]	90 [71.5-108]	90 [76.1-98.3]
Actual weight considered for nutritional calculation, kg	75.1 [64.2-85.5]	80 [66.5-84]	71.4 [62.3-84.6]	73 [68.9-81.4]	82.9 [70.2-87]	78.1 [69-82.7]
Energy intakes, kcal/day	2178 [1620-2300]	1800 [1500-2000]*	2084 [1667-2300]	1900 [1578-2100]	2150 (1800-2363]	2017 [1650-2400]
Energy intakes, kcal/kg/day	26.4 [23.6-31.4]	22.5 [19.8-26.6]*	27.6 [24-31.8]	25.7 [22.9-28.8]*	27.7 [25.7-28.8]	26.2 [22.1-31.5]
Energy healthy target (FAO/WHO/UNU), kcal/day	2670 [2327-2963]	2315 [2187-2644]*	2529 [2203-2866]	2338 [2130-2535]*	2779 [2342-2973]	2350 [2196-2250]*
Energy intakes, % of healthy target	71.7 [64.2-88.9]	73.2 [61.9-85.9]	79.3 [70.3-89.7]	79.2 [68.7-89.4]	80.1 [73-87.6]	84.2 [69.6-97.5]
Energy ICU target (25 kcal/kg/day), kcal/day	1878 [1606-2138]	2000 [1663-2100]	1784 [1559-2114]	1825 [1722-2035]	2072 [1755-2175]	1952 [1724-2066]
Energy post-ICU target (35 kcal/kg/day), kcal/day	2629 [2249-2993]	2800 [2328-2940]	2498 [2182-2960]	2555 [2411-2849]	2901 [2457-3045]	2732 [2414-2893]
Protein intakes, g/day	76.8 [50-100]	70 [50-81.7]	79.5 [63.7-90]	80 [57.3-95]	85.8 [71.1-102.5]	80.1 [64.5-112.5]
Protein intakes, g/kg/day	0.95 [0.72-1.32]	0.92 [0.65-1.12]	1.11 [0.85-1.34]	1 [0.78-1.24]	1.15 [0.97-1.31]	1.08 [0.89-1.34]
Protein healthy target (0.8 g/kg/day), g/day	60.1 [51.4-68.4]	64 [53.2-67.2]	57.1 [49.9-67.7]	58.4 [55.1-65.1]	66.3 [56.2-69.6]	62.4 [55.2-66.1]
Protein intakes, % of healthy target	118.7 [88.5-163.6]	115.4 [81-139.8]	138.4 [105.9-167.7]	126.2 [97.4-154.3]	143.3 [121.7-164.1]	134.5 [110.9-167.3]
Protein ICU target (1.3 g/kg/day or 2 g/kg/day if obese, or 0.8 g/kg/day if CKD), g/day	91.7 [73.4-122.9]	86.4[65.6-120.9]	102.7 [76.5-154.9]	89 [73.3-105.3]	123.5 [84.5-163.7]	117 [82.9-165]
Protein post-ICU target (1.5 g/kg/day or 2g/kg/day if obese or 0.8 g/kg/day if CKD), g/day	102 [82.9-139.4]	99.7 [65.6-133.5]	118.5 [88.3-154.9]	102 [84-121.5]	142.5 [97.5-163.7]	139.9 [92.4-165]
Protein intakes, % of post-ICU target	61 [42.2-95.8]	70.2 [48.6-93.1]	58.6 [47.5-84.1]	75.9 [48.7-105.3]	71.8 [47.6-90.7]	69.1 [43-107]

Data are expressed as median with lower and upper quartiles [Q1-Q3]. CKD: chronic kidney disease; ICU: intensive care unit. * significant difference ($p<0.05$) between the older adults and the younger patients.
